# The Hot Water Treatment

**Published:** 1883-11-20

**Authors:** 


### THE HOT WATER TREATMENT.

### DIRECTIONS FOR USING HOT WATER ACCORDING TO THE SALISBURY PLANS.

1. *The water must be hot; not cold or lukewarm.*—This is to excite downward peristalsis of the alimentary canal. Cold water depresses as it uses animal heat to bring it up to the temperature of the economy, and there is a loss of nerve force in this proceeding.

Lukewarm water excites upward peristalsis or vomiting, as is well known. By hot water is meant a temperature of 110 degrees to 150 degrees F., such as is commonly liked in the use of tea and coffee. In cases of diarrhœa the hotter the better. In cases of hemorrhages the temperature should be at blood heat. Ice water is disallowed in all cases, sick or well.

2. *Quantity of hot water at a draught.*—Dr. Salisbury first began with one-half pint of hot water, but he found it was not enough to wash out nor to bear another test founded on the physiological fact that the urine of a healthy babe suckling a healthy mother (the best standard of health) stands at a specific gravity varying from 1015 to 1020. The urine of the patient should be made to conform to this standard, and the daily use of the urinometer tells whether the patient drinks enough or too much hot water. For example, if the specific gravity of the urine stands at 1030, more hot water should be drunk, unless there is a loss by sweating. On the other hand, should the specific gravity fall to 1010, less hot water should be drunk. The quantity of hot water varies usually from one-half to one pint or one and a half pints at one time drinking.

The urine to be tested should be "the urina sanguinis" or that voided just after rising from bed in the morning before any meals or drinks are taken.

The quantity of urine voided in twenty-four hours should measure from forty-eight to sixty four ounces. The amount will, of course, vary somewhat with the temperature of the atmosphere, exercise, sweating, etc., but the hot water must be given so as to keep the specific gravity to the infant's standard, to wit, 1015 to 1020. The urinometer will detect at once whether the proper amount of hot water has been drunk, no matter whether the patient is present or absent. Another test is that of odor. The urine should be devoid of the rank "urinous" smell so well known, but indescribable.

The Salisbury plans aim for this in all cases, and when the patients are true and faithtul the aim is realized.

3. *Times of taking hot water.*—One hour to two hours before each meal, and half an hour before retiring to bed.

At first Dr. Salisbury tried the time of one hour before meals, but this was apt to be followed by vomiting. One hour to two hours allows the hot water time enough to get out of the stomach before the food enters or sleep comes, and thus avoids vomiting. Four times a day gives an amount of hot water sufficient to bring the urine to the right specific gravity, quantity, color, odor, and freedom from deposit on cooling. If the patient leaves out one dose of hot water during an astronomical day, the omission will show in the increased specific gravity as indicated by the urinometer, in the color, etc. Should the patient be thirsty between meals, eight ounces of hot water can be taken any time between two hours after a meal and one hour before the next meal. This is to avoid diluting the food in the stomach with water.

4. *Mode of taking the hot water.*—In drinking the hot water it should be sipped, and not drunk so fast as to distend the stomach and make it feel uncomfortable. From fifteen to twenty minutes may be consumed during the drinking of the hot water.

5. *The length of time to continue the use of hot water.*—Six (6) months is generally required to wash out the liver and intestines thoroughly.

As it promotes health the procedure can be practiced by well people throughout life, and the benefits of "cleanliness inside" be enjoyed. The drag and friction on human existence, from the effects of fermentation, foulness, and indigestible food, when removed, gives life a wonderful elasticity and buoyancy somewhat like that of the babe above alluded to.

6. *Additions to hot water.*—To make it palatable, in case it is desired, and medicate the hot water, aromatic spirits of ammonia, clover tea blossoms, ginger, lemon juice, sage, salt, and sulphate of magnesia are sometimes added. Where there is intense thirst and dryness, a pinch of chloride of calcium or nitrate of potash may be added to allay thirst and leave a moistened film over the parched and dry mucous membrane surfaces. When there is diarrhœa, cinnamon, ginger and pepper may be boiled in the water, and the quantity drunk lessened. For constipation a teaspoonful of sulphate of magnesia or one-half teaspoonful of taraxacum may be used in the hot water.

7. *Amount of liquid to be drunk at a meal.*—Not more than eight ounces. This is in order not to dilute the gastric juice or wash it out prematurely, and thus interfere with the digestive processes.

8. *The effects of drinking hot water as indicated, are* the improved feelings of the patient. The fæces become black with bile washed down its normal channel. This blackness of fæces lasts for more than six months, but the intolerable fetid odor of ordinary fæces is abated and the smell approximates the odor of healthy infants suckling healthy breasts, and this shows that the ordinary nuisance of fetid fæces is due to the want of washing out and cleansing the alimentary canal from its fermenting contents. The urine is clear as champagne, free from deposit on cooling, or odor, 1015 to 1020 specific gravity, like infant's urine. The sweat starts freely after drinking, giving a true bath from centre of body to peripher. The skin becomes healthy in feel and looks. The digestion is correspondingly improved, and with this improvement comes a better working of the machine. All thirst and dry mucous membranes disappear in a few days, and a moist condition of the mucous membrane and skin takes place. Ice water in hot weather is not craved for, and those who have drunk ice-water freely are cured of this propensity. Inebriety has a strong foe in the use of hot water.

9. *Summary of general considerations on the therapeutical drinking of hot water.*—

(*a*) Foundation of all treatment of chronic diseases.

(*b*) Excites downward peristalsis.

(*c*) Relieves spasm or colic of the bowels by applying the relaxing influence of heat inside the alimentary canal, just as heat applied outside the abdomen relieves.

(*d*) Dilutes the ropy secretions of the whole body, and renders them less adhesive, sticky and tenacious.

(*e*) Inside bath.

(*f*) Dissolves the abnormal crystalline substances that may be in the blood and urine.

(*g*) Necessary to have the hot water out of the stomach before meals.

(*h*) Use is to wash down the bile, slime, yeast and waste, and have the stomach fresh and clean for eating.

(*i*) Promotes elimination everywhere.

(*j*) If objection is made, it must be remembered that we are 75 per cent. water.

(*k*) The gas that is sometimes eructated after drinking hot water is not produced by the hot water, but was present before, and the contraction of peristalsis ejects it, or sometimes it is that the air is swallowed in sipping as horses suck air. The amount of gas contained in the alimentary canal is larger than most are aware of, and yet it is not excessive, as it takes some time to eruct a gallon of gas from the stomach. This length of time can be tested by submerging a gallon jug filled with air under water, and observing how long it will be in filling with water.

(*l*) Some physicians have advised against hot water, on the ground that it would "burn the coating of the stomach." If this is so, then a denudation of the lining of the stomach continuously for twenty-four years is compatible to a state of otherwise perfect health with no sign of illness for that period of time, and is also compatible with the numerous cases that have occurred under the use of hot water as a foundation for treatment during the past twenty-five years. Again, the same physicians drink tea and coffee at the same temperature, and this act belies their warning and shows their inconsistency and want of consideration before speaking.

(*m*) These dicta about the therapeutic drinking of hot water were founded on physiological experiments at the outset, verified in pathology and based on the experience derived from the treatment of thousands of cases since 1858. They are open, so that all who will may partake of this "water of life freely."

10. *Personal estimate of the founder of this practice.*—"If I were confined to one means of medication, I would take hot water. I have drunk it for twenty-five years."

*Corroboration of the writer.*—The writer testifies that his own personal experience and observation corroborate the truth of these statements of the Salisbury plans.—*Dr. Cutter in Gaillard's Medical Journal.*

